# Gilts not detected in estrus in a batch farrowing breeding herd: a hormonal strategy to improve gilt utilization

**DOI:** 10.1590/1984-3143-AR2025-0131

**Published:** 2026-03-20

**Authors:** Monike Willemin Quirino, Michele Dezordi Franz, Arthur Martelli, Thomaz Lucia, Fabiana Moreira, Vanessa Peripolli, Bernardo Garziera Gasperin, Rafael da Rosa Ulguim, Ivan Bianchi

**Affiliations:** 1 Instituto Federal Catarinense, Araquari, SC, Brasil; 2 Universidade Federal de Pelotas, Pelotas, RS, Brasil; 3 Universidade Federal do Rio Grande do Sul, Porto Alegre, RS, Brasil

**Keywords:** puberty, pigs, estrus, gonadotropins, altrenogest

## Abstract

This study evaluated reproductive performance of gilts classified as in anestrus in a batch farrowing system. Thirty-three gilts not expressing estrus within 40 days post-arrival were treated with chorionic gonadotropins, with blood collected for progesterone (P4) analysis on the same day. Thirteen gilts expressed estrus within 7 days, and 12 were inseminated (232.8 ± 2.1 days old), with 11 farrowing (91.7%; 14.2 ± 0.83 total piglets). Nineteen gilts had P4 ≤ 2.6 ng/mL at treatment; among the 20 non-responders, 10 had P4 > 2.6 ng/mL. A strong association was observed between P4 levels (≤ 2.6; > 2.6 and < 19; ≥ 19 ng/mL) and the responsiveness of gilts to gonadotropin treatment (*P* < 0.01; Cramér’s V = 0.63), with greater responsiveness in gilts with P4 levels ≤ 2.6 ng/mL. Non-responsive gilts (*n* = 20) received Altrenogest for 10 – 11 days, with 17 showing estrus within 10 days and 15 farrowing (88.2%; 14.3 ± 0.84 total piglets). Estrus detection failures and silent heat likely caused misdiagnosis. Gonadotropin, with or without Altrenogest, reduced culling ~8-fold, allowing 41–58% of gilts to reach at least the 3^rd^ farrowing.

## Introduction

Five to 15% of prepubertal gilts may fail to exhibit estrus within a given timeframe, often being subjected to chorionic gonadotropins administration for estrus induction (Kirkwood and De Rensis, 2016). Nevertheless, this protocol’s efficiency also depends on the occurrence of false anestrus (Knox, 2016). Additionally, gonadotropins in gilts incorrectly identified as anestrus can also lead to abnormal estrous cycles (Estienne and Crawford, 2015; Brito et al., 2024).

Gilts failing to respond to gonadotropins treatment could be alternatively supplemented with Altrenogest, which allows the spontaneous luteolysis in gilts misdiagnosed as in anestrus (Stančić et al., 2016). This approach may be a valuable strategy, especially for batch farrowing breeding herds; however, limited data are available on the field effectiveness of these strategies in promoting estrus expression and synchronization to allow timely insemination along with the breeding batch.

This observational study assessed the reproductive performance of gilts not detected in estrus in a batch farrowing system that were treated with chorionic gonadotropins, followed or not by Altrenogest supplementation according to their responsiveness to the estrus induction protocol.

## Methods

Procedures were approved by the Institutional Committee for the Ethical Use of Animals of the Instituto Federal Catarinense (439/2023) and conducted according to the guidelines of the Brazilian College of Animal Experimentation.

### Animals and facilities

The 33-week study in a piglet unit (26°46'06.4"S, 52°43'46.8"W) involved gilts (TN70^®^) not detected in estrus within 40 days post-arrival. Estrus was checked twice daily using the back-pressure test with boar contact. Gilts were housed in collective pens (1.0 m^2^/gilt) with slatted floors, fed 2.5 kg/day of gestation diet, then moved to individual pens (0.6 × 2.2 m) 14 days before breeding, receiving 4 kg/day. Two days after the last insemination, they were transferred to gestation pens (2.24 m^2^/gilt) fed 1.9 – 2.3 kg/day until day 108, then moved to farrowing crates (1.60 × 2.70 m). Water was always available.

### Estrus induction, blood collection, and Altrenogest treatment

Thirty-three gilts not showing estrus within 40 days post-arrival received 400 IU eCG + 200 IU hCG at 208.2 ± 7.2 days old (i.m., day 41; Gestavet 600^®^; Biogénesis Bagó, China). Blood was collected for serum progesterone (P4) analysis; gilts with P4 ≤ 2.6 ng/mL were classified as non-cyclic (Chung et al., 2002). Estrus was checked twice daily for 7 days post-treatment and again 15 days later. Gilts responsive to gonadotropin treatment were inseminated in the following estrus (non-induced estrus).

**Figure 1:**
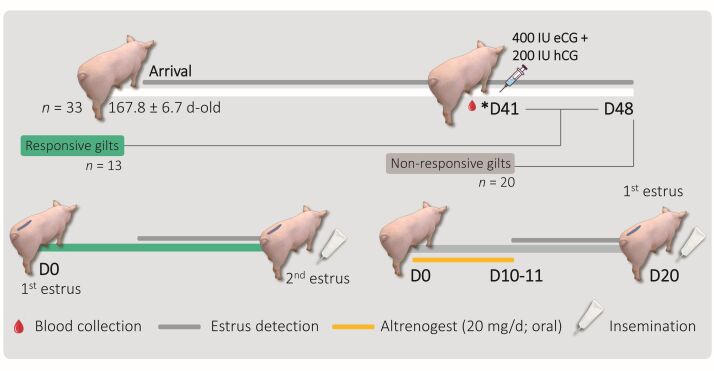
Scheme illustrating the study methodology. D41: 41 days post-arrival. Blood collection: to assess serum progesterone.

Twenty gilts not showing estrus within 7 days after gonadotropin administration received Altrenogest (20 mg/day orally) for 10 – 11 days, starting 7 days post-gonadotropin treatment (**Figure 1**). Estrus was checked twice daily after finishing Altrenogest supplementation.

All gilts in estrus were assessed regarding their body condition score and caliper units and were inseminated immediately or 12 h later, with additional inseminations every 24 h. Pregnancy was confirmed ~28 days by ultrasonography (Sono V5+, SonoWin Science Technology Inc., China).

Farrowing performance across the first three farrowings was monitored for all gilts. For comparison purposes, the farrowing performance of 11 contemporary gilts that exhibited their first estrus within 40 days post-arrival (therefore not treated with gonadotropin) was also recorded. These non-treated gilts were inseminated at their fourth estrus (according to the farm breeding schedule) at the same age as gilts treated with gonadotropin or gonadotropin plus Altrenogest, and were randomly selected from the same batches as the treated gilts.

### Data analysis

Data was analyzed using SAS^®^. P4 levels of responsive and non-responsive gilts were compared with the Wilcoxon test. Associations between P4 classes and estrus responsiveness were assessed by Chi-square or Fisher’s exact test, using the contingency coefficient (CC) or Cramér’s V.

### Results

**Figure 2:**
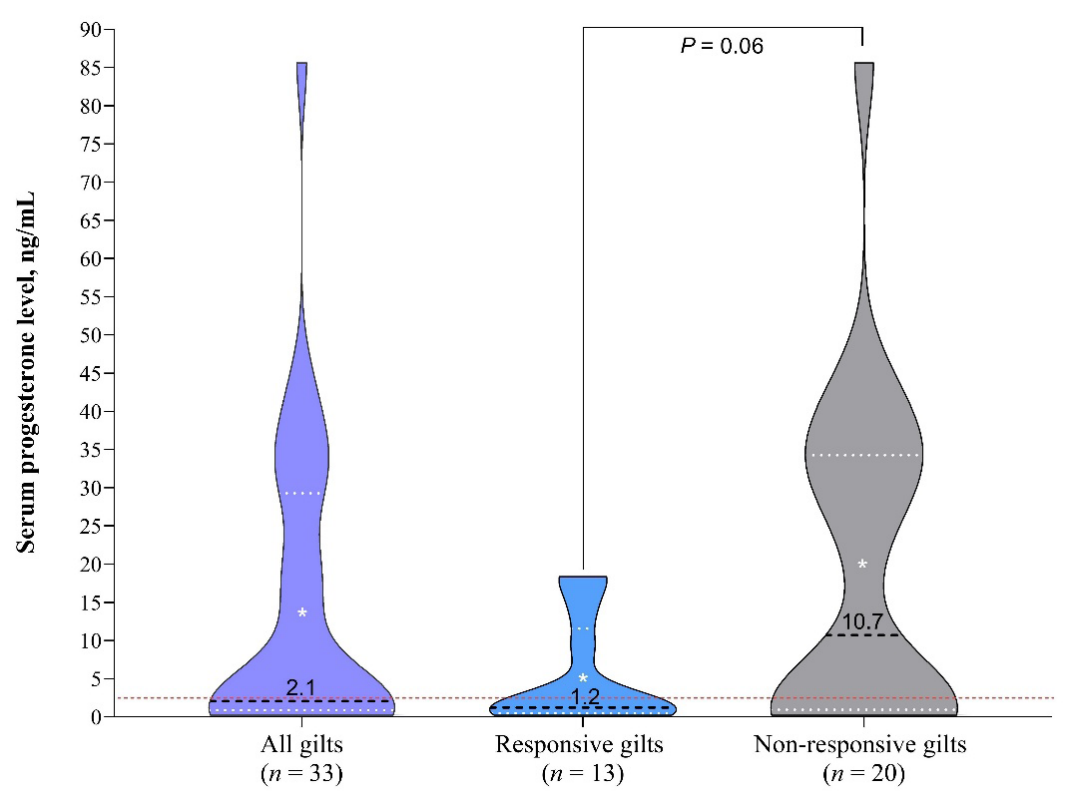
General data on serum progesterone (P4) in responsive and non-responsive gilts to gonadotropin treatment after not being detected in estrus within 40 days post-arrival. Red dotted lines: P4 concentration limit (≤ 2.6 ng/mL) for considering gilts as non-cyclic. Black dotted line: median. White asterisk: mean.

**Figure 3:**
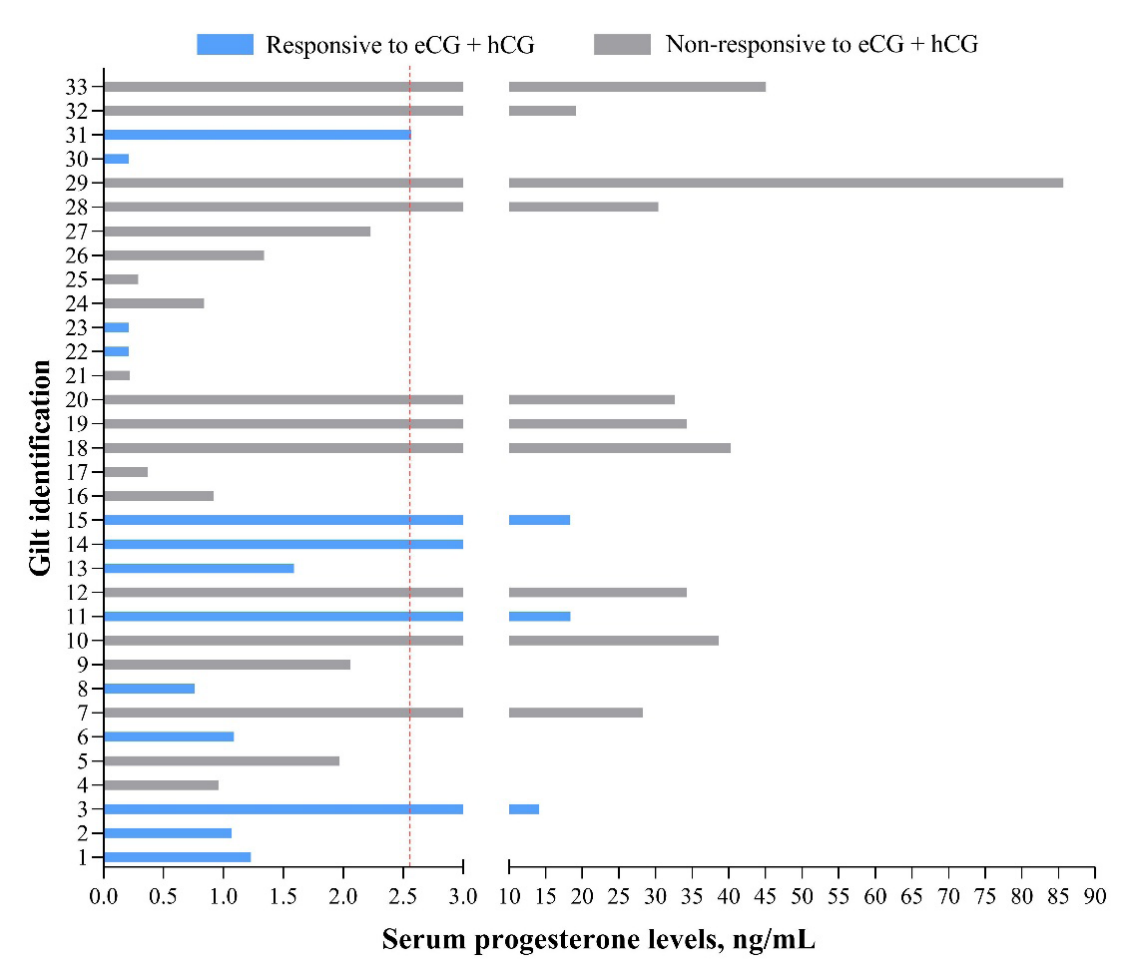
Individual serum progesterone (P4) levels in responsive and non-responsive gilts to gonadotropin treatment after not being detected in estrus within 40 days post-arrival.

**Figure 4:**
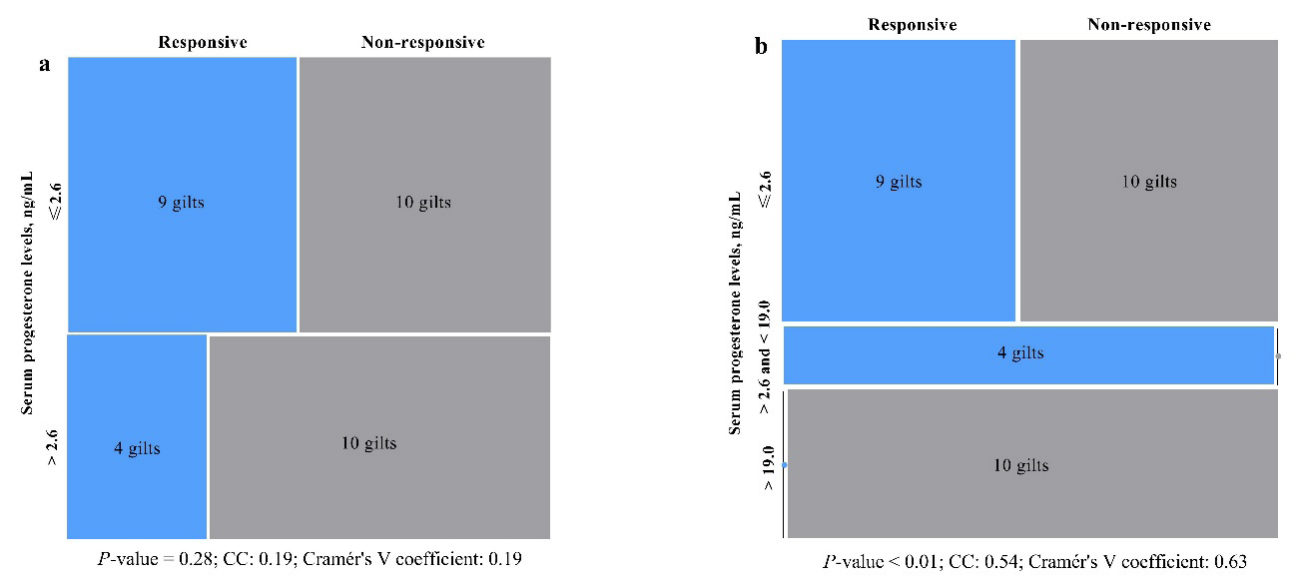
Measures of association between serum progesterone (P4) levels and gilts’ responsiveness to chorionic gonadotropins, considering two **(a)** or three **(b)** classes for P4. CC: contingency coefficient.

Of 33 gilts treated with gonadotropins, 13 (39.4%) showed estrus within 7 days post-treatment, but one gilt did not show a second estrus; therefore, 12 were inseminated (~ 232 days old), and 11 farrowed (91.7%; 14.2 ± 0.83 total, 13.8 ± 0.76 born alive; **Tables 1** and **2**). At gonadotropin treatment, 57.6% of gilts had P4 levels ≤ 2.6 ng/mL. Responsive gilts (*n* = 13) tended to have lower P4 levels (median 1.2 ng/mL) than non-responsive gilts (*n* = 20; median 10.7 ng/mL; *P* = 0.06). Among non-responsive gilts, 50% had P4 levels ≤ 2.6 ng/mL. All the remaining non-responsive gilts with P4 levels > 2.6 ng/mL presented levels ≥ 19.0 ng/mL (**Figures 2** and **3**). No association between P4 levels and responsiveness to gonadotropins treatment was found when considering P4 levels as ≤ 2.6 or > 2.6 ng/mL (*P* = 0.28), but a three-level classification (≤ 2.6; > 2.6 and < 19; ≥ 19 ng/mL) revealed a strong association (*P* < 0.01; Cramér’s V coefficient: 0.63; **Figure 4**).

**Table 1:** Carachteristics and farrowing rate of gilts not detected in estrus* and treated with equine and human chorionic gonadotropin (eCG+hCG; *n*=33) followed or not by Altrenogest.

	**Gilts responsive to eCG+hCG** (*n* = 13)	**Gilts treated with Altrenogest** (*n* = 20; non-responsive to eCG+hCG)
% of gilts in estrus within 7 d post-gonadotropins	39.4 (13/33)	.
Interval gonadotropins and estrus, d	3.5 ± 1.7	.
Age at 1^st^ estrus post-gonadotropins, d	211.8 ± 6.0	.
Second estrus post-gonadotropin, % of gilts	92.3 (12/13)	.
Interval 1^st^ and 2^nd^ estrus, d	20.3 ± 2.0	.
Interval gonadotropins and Altrenogest, d	.	7.4 ± 0.7
Age at Altrenogest treatment onset, d	.	214. ± 7.7
% of gilts in estrus within 7 d post-Altrenogest	.	85.0 (17/20)
End of Altrenogest treatment and estrus, d	.	6.8 ± 1.0
Inseminated gilts, %	36.4 (12/33)	85.0 (17/20)
Age at breeding, d	232.1 ± 7.0	233.1 ± 7.8
Body condition score at breeding	4.2 ± 0.5	4.0 ± 0.5
Caliper at breeding	16.6 ± 1.7	16.5 ± 1.7
Farrowing rate, %	91.7 (11/12)	88.2 (15/17)

* Within 40 days post-arrival. Data: percentage (%; n/n) or mean ± standard deviation.

**Table 2:** Descriptive data on farrowing performance across three parities for gilts responsive to gonadotropin treatment (administered when gilts were not detected in estrus within 40 days post-arrival) and non-treated gilts (detected in estrus within 40 days post-arrival).

**Response**	**Group**	**1^st^ farrowing**	**2^nd^ farrowing**	**3^rd^ farrowing**
Total piglets born	Non-treated gilts	15.9 ± 0.73	16.4 ± 0.66	15.4 ± 1.58
	Gonadotropin	14.2 ± 0.83	14.0 ± 1.49	17.2 ± 0.77
	Gonadotropin + Altrenogest	14.3 ± 0.84	14.7 ± 0.82	14.0 ± 1.63
Piglets born alive	Non-treated gilts	15.3 ± 0.55	14.4 ± 1.37	15.0 ± 1.07
	Gonadotropin	13.8 ± 0.76	13.8 ± 1.51	16.4 ± 0.74
	Gonadotropin + Altrenogest	14.1 ± 0.82	13.5 ± 1.00	13.5 ± 1.43
Stillborn	Non-treated gilts	0.33 ± 0.19	2.09 ± 1.53	1.09 ± 0.71
	Gonadotropin	0.36 ± 0.27	0.10 ± 0.09	0.80 ± 0.37
	Gonadotropin + Altrenogest	0.20 ± 0.14	0.27 ± 0.19	0.50 ± 0.25
Mummified	Non-treated gilts	0.27 ± 0.26	0.00 ± 0.00	0.00 ± 0.00
	Gonadotropin	0.00 ± 0.00	0.10 ± 0.09	0.00 ± 0.00
	Gonadotropin + Altrenogest	0.00 ± 0.00	0.90 ± 0.87	0.00 ± 0.00

Data: mean ± standard error of the mean.

Gonadotropin group: gilts responsive to eCG+hCG (11 gilts had a 1^st^ farrowing and 10 gilts had 2^nd^ and 3^rd^ farrowing, since one female was discarded on the day of its first weaning).

Gonadotropin + Altrenogest: gilts non-responsive to gonadotropin that were treated with Altrenogest for 10 – 11 days (20 mg/day), starting 7 days post-gonadotropin (15 gilts had a 1^st^ farrowing, 11 gilts had a 2^nd^ farrowing, and 8 gilts had the 3^rd^ farrowing).

Non-treated gilts: no hormone treatment (11 gilts that had a 1^st^, 2^nd^, and 3^rd^ farrowing).

Seventeen of 20 gilts receiving Altrenogest, after not being detected in estrus within 7 days post-gonadotropin, showed estrus within 10 days after supplementation. All 17 gilts were inseminated (233 days old) and 15 gilts farrowed (88.2%; 14.3 ± 0.84 piglets born and 14.1 ± 0.82 piglets born alive; **Tables 1** and **2**). Descriptive data on farrowing performance across three parities for gilts responsive and non-responsive to gonadotropin treatment as well as randomnly contemporary non-treated gilts (detected in estrus within 40 days post-arrival and inseminated at ~ 232 days old) are shown in **Table 2** (*n* = 11).

## Discussion

Overall, our results showed that estrus response to gonadotropins was low (39%), whereas most non-responsive gilts exhibited estrus within seven days after Altrenogest (85.0%). Moreover, the hormonal strategy appproched in this study reduced culling rates nearly eightfold and allowed 41 to 67% of inseminated gilts reaching at least the 3^rd^ farrowing.

Estrus induction with gonadotropins is common in gilts, with ~70% expected to show estrus within 3–7 days (Knox, 2016). Here, limited eCG+hCG effectiveness may reflect that some gilts were already pubertal, as 50% of non-responders (10/20) had P4 > 2.6 ng/mL (mid-luteal phase, 28.2–85.7 ng/mL). Four of 13 responsive gilts also had P4 > 2.6 ng/mL, but their P4 levels (9.0–18.3 ng/mL) suggest they were likely undergoing luteolysis, as estrus occurred 3 to 5 days later (Chung et al., 2002). No association was found between estrus response and P4 (≤ vs. >2.6 ng/mL), possibly because some non-responsive gilts had recently ovulated, showing low P4 despite lack of response. Future studies including estrogen measurement, follicular monitoring by ultrasound evaluation (especially before the gonadotropin treatment onset), and paired P4 sampling to improve cycle classification and induction strategies must be performed.

Stančić et al. (2016) suggested Altrenogest can synchronize cyclic gilts misclassified as anestrus or induce puberty in prepubertal ones. In our study, 85% of gonadotropin-non-responsive gilts showed estrus within 7 days. Nine gilts had P4 ≥ 2.6 ng/mL at gonadotropin administration, indicating luteal phase, while eight had P4 ≤ 2.6 ng/mL. Progestogens may increase LH pulses by reducing estrogen feedback (Mani and Blaustein, 2012), but in pigs, it does not seem to enhance boar-induced estrus (Baldessar et al., 2023). Thus, these gilts may have recently ovulated, explaining low P4, lack of gonadotropin response, and later synchronization with Altrenogest.

Our results also highlight the need for accurate estrus detection. In a Brazilian herd, 30–40% of culled gilts were misdiagnosed (Baldessar et al., 2023). In our study, anestrus rate was ~5% (33/636), with 45% showing P4 ≥ 2.6 ng/mL, indicating misclassification mainly from detection failures. Due to puberty complexity and diagnostic challenges, technician training is essential, but rapid P4 testing with two samples 7 days apart may also contribute to reducing misdiagnosis and optimizing hormone use (Chung et al., 2002; Marques et al., 2023). Additionally, Altrenogest treatment in non-responsive gilts showed that supplementation can reduce culling and integrate these gilts into breeding herds effectively.

Using chorionic gonadotropins, with or without progestogen based on response, may optimize replacement female use and contribute to herd productivity. In this study, culling decreased nearly eightfold (5%–0.6%), offering potential financial benefits in herds with high anestrus rate. Moreover, farrowing rates were 88.2–91.7% with 14.2 – 14.3 total piglets compared to 100% with 15.9 total piglets from contemporary gilts showing spontaneous estrus within 40 days post-arrival. For all gilts showing spontaneous estrus over the whole monitored period, these responses were 97.1% and 15.3 piglets (~ 500 gilts; data not shown). Of gilts inseminated after gonadotropin response, 58% (7/12) reached the 3^rd^ farrowing without returning to estrus or being discarded after weaning, averaging 17.2 piglets born, while 41% (7/17) of gilts after gonadotropin + Altrenogest reached the 3^rd^ farrowing (with no returning to estrus or being discarded after weaning), averaging 14.0 piglets. Still, larger studies are needed, and hormonal treatments should be used cautiously, considering cost-benefit and potential reproductive issues, as true anestrus may have genetic causes (Quirino et al., 2025).

## Conclusion

Estrus detection failures accounted for most misclassified anestrus cases, reducing the response to gonadotropin induction (39%). Altrenogest induced estrus in 85% of non-responders, lowering culling nearly eightfold. Repeated rapid P4 testing could improve management decisions and support more strategic hormone use.
